# Building a Collaborative Translational Research Platform: Identifying Barriers and Enablers From Basic Research to Primary Healthcare

**DOI:** 10.7759/cureus.78699

**Published:** 2025-02-07

**Authors:** Jean-Sebastien Paquette, Julie-Alexandra Moulin, Andréanne Sheehy, Gardy Lavertu, Ella Diendéré, Alfred Kodjo Toi, Marie-Claude Tremblay, Étienne Audet-Walsh, France Légaré, Caroline Rhéaume, Virginie Blanchette, Patrick Archambault, Jean-Pierre Després, Léanie Moreau, Joanie Neveu, Léanne Day Pelland

**Affiliations:** 1 Family Medicine and Emergency Medicine, Université Laval, Quebec City, CAN; 2 Research, VITAM - Centre de Recherche en Santé Durable, Quebec City, CAN; 3 Psychology, Université du Québec à Trois-Rivières, Trois-Rivières, CAN; 4 Psychology, Université de Montréal, Montreal, CAN; 5 Research, Centre Hospitalier Universitaire (CHU) de Québec - Université Laval, Quebec City, CAN; 6 Molecular Medicine, Université Laval, Quebec City, CAN; 7 Human Kinetics and Podiatric Medicine, Université du Québec à Trois-Rivières, Trois-Rivières, CAN; 8 Kinesiology, Université Laval, Quebec City, CAN; 9 Research, Centre Intégré de Santé et de Services Sociaux de Lanaudière, Saint-Charles-Borromée, CAN; 10 Faculty of Medicine, Université Laval, Quebec City, CAN

**Keywords:** barriers, collaboration, enablers, focus groups, qualitative research, research-to-practice continuum, translational research

## Abstract

Introduction

The capacity to translate basic research discoveries into clinical applications and to synthesize, disseminate, and integrate clinical research results into practice remains challenging. To help innovate the means of communicating and disseminating knowledge between actors across the research-to-practice continuum, this study aims to identify barriers and enablers in building and implementing a collaborative platform that will bring together all the actors involved.

Methods

The study was conducted based on a qualitative descriptive design and a deductive thematic analysis. Recruitment was performed using a purposive sampling strategy. Data were collected through three focus groups with a total of 23 participants involving actors from each pillar of the research-to-practice continuum: eight basic researchers (Group 1), eight clinical and organizational researchers (Group 2), and seven knowledge users, including healthcare professionals and patient partners (Group 3).

Results

Few participants had concrete experience in the field of translational research, but half of them had already collaborated with actors from other research pillars. Identified barriers (e.g., length and complexity of the process, differences in knowledge and professional goals between clinical and basic research, insufficient resources and time to invest in research projects, lack of recognition of the added value of patient implication) and enablers (e.g., use of clear guidelines and targeted research questions, networking by matching according to area of practice and interests, introduction to research in the curriculum of medical students, dissemination of scientific information in a language understandable to all) emerging from the focus groups were clustered into four main categories: (i) translational research project concretization, (ii) basic research applicability, (iii) clinician availability and commitment, and (iv) patient involvement and recruitment. These barriers and enablers emphasized the need to decomplexify the translational research process for all actors involved in the research-to-practice continuum. They also accentuated the need to recognize the social responsibility of basic research and increase its impact on the ground, intensify the exposure of medical students to research and value clinicians’ involvement in research activities, and engage patients as research partners to help prioritize research topics and communicate science comprehensibly. The success indicators of the main platform would be the relevance, strength, and duration of collaborations, the number of projects implemented and completed, and the grants and funding obtained.

Conclusion

We identified key barriers and enablers to implement a functional and dynamic collaborative platform. Participants’ desire to collaborate combined with the absence of a tool to foster efficient collaborations are indicators of the importance of our approach. Creating a readily accessible collaborative platform to all actors across the research-to-practice continuum has the potential to drive research advances based on the needs of patients, clinicians, and the public, and thus facilitate their clinical application in a timelier manner to improve healthcare standards and services as well as population health outcomes.

## Introduction

Background

For quite some time now, collaborative research has proven its worth in strengthening health systems by improving disease management and access to high-quality, appropriate, and cost-effective care [1-3]. A more widely used collaborative approach since the turn of the 21^st^ century, translational research is a scientific approach that aims to translate research findings into clinical interventions such as treatments, diagnostics, or prevention strategies by bridging the gaps between scientific discoveries and their practical application to improve human health [4,5]. The translation of research findings into practice is predominantly viewed as a linear process of successive steps that roughly begins with discoveries from basic scientific research (Step 1), leading to clinical research in humans (Step 2) before being applied in clinical practices (Step 3) [5]. However, the flow of information throughout this process is impeded by two major gaps. The first gap refers to the reduced capacity to translate the results of discoveries generated by basic science into human clinical research, also known as the “bench-to-bedside” phase. The second gap pertains to the limited capability to synthesize, disseminate, and integrate clinical research results more broadly into healthcare decision-making and clinical practice (called the “bedside-to-practice” phase) [6]. In fact, less than 15% of research evidence is successfully produced and reported to serve advances in healthcare [7] and it takes an average of 17 years for these to reach the last phase of the research-to-practice continuum [8]. To ensure scientific advances are more effectively implemented in real-world settings, it is critical to rethink collaboration modalities and develop innovative ways to communicate and disseminate knowledge seamlessly between the different actors of the research-to-practice continuum, namely basic researchers (pillar 1), clinical researchers (pillar 2) and knowledge users, including healthcare providers, patients, and the public (pillar 3).

Context of the study

Prior to this study, our team conducted a scoping review to identify and describe collaborative models that connect biomedical research with primary healthcare practice in chronic disease management [9]. The review revealed a significant lack of literature on the subject, retaining only 13 studies describing 20 unique collaborative models implemented between 1967 and 2014 (median = 2010). Of these 20 collaborative models selected, only five included all the actors of the research-to-practice continuum, i.e., biomedical researchers, clinical researchers, primary healthcare providers, and patients, citizens, or users. Seven models reported a measurable impact such as the creation of a strong base of talented expert researchers, the participation of patients in workshops, and the enrollment of medical professionals in training programs. Common collaboration barriers (lack of financial support, knowledge, leadership, and robust management structure) and enablers (partnership, cooperation, multidisciplinary research teams, and project management) were also identified from these models. This review subsequently led to the development of a logic model (Appendix A) that provided guidelines and insights for health research decision-makers while implementing optimal translational collaboration in primary care research related to chronic disease management [10].

Published studies on collaboration models in chronic disease management through translational research are scarce and fail to provide sufficient details on the modalities to prioritize to create a suitable collaborative platform. Using the experiential knowledge of the actors in the research-to-practice continuum, the present study aims to further identify collaboration barriers and enablers to build and implement an innovative, effective, and sustainable collaborative platform that will bring together all the actors involved.

## Materials and methods

Study design and approach

The was a qualitative descriptive study conducted based on the consolidated criteria for reporting qualitative research (COREQ) [11], using the focus group methodology. Focus groups are widely used in health research to obtain information by engaging a small group of individuals to interact and share their experiential knowledge in the presence of a facilitator [12,13]. The study included three focus groups, one for each pillar of the research-to-practice continuum, to capitalize on the shared experiences of individuals within the same pillar while bringing together a range of professions to maximize the exploration of different perspectives within each group setting.

Participants

The recruitment of participants was performed using a purposive sampling strategy, which involves a conscious selection of individuals, on a voluntary basis, with the appropriate experiences or characteristics [14]. In this case, participants had to represent the actors of the different pillars of the research-to-practice continuum. Participants were identified according to the nature of their work: basic researchers working in a laboratory whose work consists of studying the molecular and physiological mechanisms of pathologies (pillar 1), clinical and organizational researchers who work with patients or on organizational interventions (pillar 2), and knowledge users including health professionals who treat patients without being involved in research and patient partners (pillar 3). Participants were recruited from the Faculty of Medicine and the Faculty of Nursing of Université Laval (Quebec City, Canada),Université de Sherbrooke (Sherbrooke, Canada), and the Groupe de Médecine de Famille Universitaire du Nord de Lanaudière (GMFU-NL) (Saint-Charles-Borromée, Canada) through various communication channels (emails, newsletters). The Learning Help System Support Unit of Quebec (Longueuil, QC, Canada) was also solicited to assist in the recruitment of patient partners. These sites were chosen because they are part of our clinical and research network.

The maximum number of participants per focus group was set at eight individuals to align with the recommendations for focus group research [15,16]. Each focus group was composed as follows: eight basic researchers from various fields such as oncology, genomics, biology, and nanomedicine (Group 1), eight clinical and organizational researchers in the fields of primary care and services, population health, and health information technologies (Group 2), and seven knowledge users, including two specialist physicians, two family physicians, one nurse, one nutritionist and kinesiologist, and one patient partner (Group 3), for a total of 23 participants. One patient partner was unable to attend the Group 3 focus group due to health issues. All participants provided written consent and the patient partner received a financial compensation. Participants’ demographic characteristics (age, gender, education, profession, years of practice, etc.) were also collected.

Research team

The research team included the principal investigator of the study who is a clinical researcher and family physician collaborating with basic researchers in different research projects (JSP), a research coordinator (AKT), two research professionals (ED and JAM), and two master’s students in psychology (AS and GL). They ensured the completion of the various stages of the study conduct as well as data collection and analysis. Focus groups were facilitated by the principal investigator (JSP) and two observers attended each meeting (AKT and ED or GL).

Data collection

Prior to the conduct of the study, the research team developed a facilitation guide to promote exchanges and interactions between group members (Appendix B) [17]. The focus groups were held from March to May 2022 and were conducted virtually via the communication platform Zoom (Zoom Communications, Inc., San Jose, California, United States). Before each focus group, features available within the virtual platform were activated to ensure security and confidentiality (waiting rooms, locked access using passwords, and participation by domain where only authenticated participants could access the focus groups). Each focus group began with a general introduction to the study, an overview of the study objectives, and data privacy considerations. Discussion topics were then presented around various themes [18]: collaboration experiences, objectives and outcomes of translational research projects, barriers and enablers of a collaborative platform, and success indicators of such a platform (Appendix C). Participants were invited to speak in turn. Each focus group duration was about two hours. Discussions were led in French and recorded using a digital voice recorder. Following the focus group, participants were invited to answer a short questionnaire to assess their level of satisfaction with the activity.

Data analysis

The discussions were transcribed verbatim by a team member, and transcripts were verified by another team member to ensure accuracy. All transcripts were analyzed using NVivo R1/2020 version 20.7.1 (Lumivero, LLC, Denver, Colorado, United States). Two team members conducted a deductive thematic analysis of the data (AS and JAM) [19]. The initial codes were generated and then associated with themes relevant to the barriers and enablers of a collaborative translational research platform. Coding differences were resolved by consensus.

## Results

Participant characteristics

Table 1 outlines the general characteristics of the participants. Participants in Group 3 were slightly younger on average than those in groups 1 and 2, with participants in Group 2 being the oldest on average. In addition to representing varied disciplines, the years of practice of the participants were broad, ranging from one year in Group 3 to 30 years in Group 2. Equal numbers of women and men were included in the study, but they were not evenly distributed within each focus group. The number of women was higher in groups 2 and 3, while only men participated in Group 1. Few participants had concrete experience in the field of translational research, but half of them had already collaborated with actors from other research pillars, with Group 3 having the highest collaboration experience.

**Table 1:** Participant characteristics Years of practice are counted in number of years of research collaboration for the patient partner.

Group	Age (years)	Years of practice	Gender		Collaboration experience		Total
	Mean ± SD	Range	Woman	Man	Yes	No	
1- Basic researchers	45 ± 5	7 – 20	0	8	3	5	8
2- Clinician and organizational researchers	48 ± 8	3 – 30	6	2	4	4	8
3- Knowledge users	40 ± 8	1 – 20	5	2	5	2	7
Total	-	-	11	12	12	11	23

Objectives and outcomes of translational research

Most participants across the three focus groups manifested their interest in translational research and found the research project relevant. They all believed that the primary objective of translational research projects was to promote the integration and active collaboration of all research pillars, leading to a positive effect on the respective environments of the actors involved. Through this process, groups 1 and 2 also aspired to raise awareness and educate patients about research findings, and groups 1 and 3 sought to contribute to the dissemination of knowledge and stay current with research advances. Groups 2 and 3 were particularly interested in building a common vision for the different environments and adding value to interventions.

Barriers and enablers of a collaborative platform

Four main categories of barriers and enablers emerged from the focus groups to implement a collaborative translational research platform: (i) translational research project concretization, (ii) basic research applicability, (iii) clinician availability and commitment, and (iv) patient involvement and recruitment. Table 2 outlines the main barriers and enablers within each category, expressed in at least two out of the three focus groups, as well as possible solutions to make enablers more tangible.

**Table 2:** Barriers, enablers, and possible solutions to implement a collaborative platform Focus group number(s) identified in parentheses.

Barriers	Enablers	Possible solutions
Translational research project concretization		
Length and complexity of the process (1,2,3)	Focus on efficiency, simplicity, and accessibility (1,2,3)	Conduct a needs analysis to ensure efficiency prior to implementation (1)
Lack of governance (structure and coordination) (1,2,3)	Use of clear guidelines and targeted research questions (1,2,3)	Structure and coordination by higher authorities and dedicated liaison officers (1,2)
Poor communication and dissemination of knowledge (1,2,3)	Creation of dedicated spaces (physical or virtual) to facilitate exchanges (1,2,3)	Integrate a discussion forum and schedule needs-specific meetings in advance (2,3)
Basic research applicability		
Differences in knowledge and professional goals between clinical and basic research (1,2,3)	Networking by matching according to area of practice and interests (1,2,3)	Submit basic research ideas to a bank of clinicians who have the desired expertise and interest (1)
Lack of awareness of basic research significance (1,3)	Promotion and advertisement of basic research discoveries (1,2)	Create and broadcast promotional capsules or videos of research work (1)
Lack of clinical relevance of basic research work (2,3)	Dissemination of concerns expressed by clinicians and patients on the ground (2,3)	Survey patients and clinicians on research questions they judge important (2,3)
Clinician availability and commitment		
Lack of resources and time to invest in research projects (1,2,3)	Funding/financial incentives for research tasks performed by clinicians (1,2,3)	Dedicate a percentage of time or salary to research work (1)
No clear added value of research (1, 2, 3)	Introduction to research in the curriculum of medical students (1,2)	Train first-year medical students in the laboratory (1)
Lack of recognition of the importance of research (2,3)	Organizational culture favoring research (1,2)	Implement research scholarships for medical students (1)
Patient involvement and recruitment		
Lack of recognition of the added value of patient implication (1,2,3)	Promotion of patient involvement in collaborations (1,2,3)	Develop projects that directly affect patients (2,3)
Difficulty in accessing patients (1,2)	Implementation of effective patient recruitment strategies (1,2)	Recruit via family physicians and family medicine groups interested in research (1,2)
Lack of popularization of scientific language (1,3)	Dissemination of scientific information in a language understandable to all (1,3)	Simplify scientific concepts using patient input (3)

Below is an overview of the four emerging categories with characteristic quotations retrieved from the transcripts and translated from French to English.

Category 1: Translational Research Project Concretization

Project concretization through translational research concerned all groups due to the length and complexity of the process, the lack of governance, and poor communication and dissemination of knowledge.

“*Every year, we make calls for integrative projects that go from basic research to the patient's bedside, and we have a lot of difficulty finding projects that have all of this spectrum. Just the understanding of what it really is, it’s not understood the same by everyone.*” ~ Group 2

“*The structure is not facilitating with all the pressure to publish and renew our grants. You say to yourself, I don't have the time to put six months into this, so instead of looking for expertise here, you'll look for it outside.*” ~ Group 1

“*I always tell myself it's a shame we don't have more sharing rounds, communication between clinicians, clinical researchers and basic researchers because we don't know what people are doing in their yard.*” ~ Group 2

As enablers, participants recommended that the platform focus mainly on efficiency, simplicity, and accessibility, with the use of clear guidelines and targeted research questions, and the creation of spaces to facilitate dynamic exchanges.

“*What is extremely important is ease [of use]. Like a platform that would allow to target your needs and that people could access by taking the metro or drinking their coffee in the morning.*” ~ Group 1

“*I think that having spaces or moments for communicating where primary care groups and basic researchers can meet and make connections could be good.*” ~ Group 3

“*I think it is also about establishing a platform in which the objectives are clear (...) with a defined framework where we know where we are going.*” ~ Group 3

Possible solutions raised by Group 1 included the conduct of a preliminary needs analysis to ensure the platform meets everyone's requirements. For governance, groups 1 and 2 proposed that the platform be led by higher authorities (e.g., board of directors) and dedicated liaison officers such as clinicians trained in basic research. To facilitate communication, groups 2 and 3 suggested inserting online forums and scheduling meetings in advance according to fields of practice, with predetermined and clear agendas.

Category 2: Basic Research Applicability

Questions raised on basic research applicability pertained to differences in knowledge and professional goals between clinical and basic research, the lack of awareness of the significance of basic research, and the perceived clinical irrelevance of basic research work.

“*I observe the same thing within our faculty where about half of the people are in basic research and half in clinical research who really have difficulty communicating and understanding each other.*” ~ Group 2

“*Basic research is not sold at all in the medical field, even at the physician level, I mean, for many people it is completely abstract.*” ~ Group 1

“*Yes, there is the time constraint, but it is also the clinical application side of the basic research ideas. When you are not in everyday practice, you do not know how it could be applied or what direction could be interesting.*” ~ Group 3

“*I think that recognition is probably a major point also for basic researchers, because they have the impression that all the work and effort they put into research is never used.*” ~ Group 3

To alleviate these barriers, participants suggested creating networks by matching based on areas of practice and interests, advertising basic research discoveries, and disseminating the concerns expressed by clinicians and patients on the ground.

“*Ultimately, it's a matchmaker we're trying to make between the basic researcher and the clinician, to have something where we can quickly see the expertise, who is interested, what the clinical trial ideas are, and what are the means to do it too.*” ~ Group 1

“*The idea is to value discoveries, there are often things happening next to us that we are not aware of.*” ~ Group 2

“*If we want to involve clinicians more, we should seek and increase the dissemination of their concerns on the ground to see if there is a possible connection with basic researchers.*” ~ Group 2

“*I think that it is important to know the daily life, the realities, and the condition in which the basic research will apply.*” ~ Group 3

As possible solutions, Group 1 proposed submitting their research ideas to a bank of clinicians who already have the desired expertise and interest, and advertising their work through promotional capsules or videos. To ensure the relevance of basic research work, Groups 2 and 3 underlined the importance of surveying patients and clinicians directly on research questions they judge important.

Category 3: Clinician Availability and Commitment

The main barriers to clinician availability and commitment are related to insufficient resources and time to invest in research projects, as well as unclear added value and lack of recognition of the importance of research.

“*Sometimes clinicians would like to put more time [into research] but they are so busy with the clinical tasks that they cannot.*” ~ Group 1

“*I don't see the relevance at this stage (...) I see the patient clearly, but basic research is not at all about the same thing. (...) I don't see the added value in getting to know each other, trying to see the bridges and the synergies to develop research.*” ~ Group 2

“*An obstacle that I see is the lack of recognition of the importance of research, you know, if the environment does not recognize research, the ministry puts pressure on you. At the clinic level, it is difficult to get involved or free up time and resources since it is the last thing that matters for your colleagues, your establishment, and the ministry.*” ~ Group 3

To address these hurdles, participants raised the need for financial incentives for research tasks performed by clinicians, the introduction of research into the curriculum for medical students to increase their knowledge in this field, and the development of an organizational culture that supports and values research.

“*It's always good to have incentives that allow physicians to have at least some recognition for the time they spend doing research.*” ~ Group 1

“*I think that the medical faculty could promote the link between physicians and the laboratory. This must happen at the start of the training. (…) It is up to the medical faculty to create this enthusiasm for research.*” ~ Group 1

“*Health professionals who have mandatory continuing education hours could interact more with basic researchers to increase exchanges.*” ~ Group 2

Participants in Group 1 advanced interesting possible solutions, such as dedicating a percentage of time or salary to clinical research work, implementing research scholarships for medical students, and training as many first-year medical students as possible in the laboratory.

Category 4: Patient Involvement and Recruitment

According to participants, the lack of recognition of the added value of patient implication in research projects, the difficulty in accessing patients, and the use of insufficiently popularized scientific language were detrimental to patient involvement and recruitment.

“*We remain relatively far from the patient. The closest we are to humans is often via samples that have been collected by certain experts.*” ~ Group 1

“*I see a lot of barriers: the lack of communication, the lack of bodies that facilitate these connections, the lack of patient involvement and recognition of their ability to be able to identify what could be of interest.*” ~ Group 2

“*The basic problem is the extraordinary difficulty in recruiting patients and identifying them. From experience I can tell you that to recruit a patient for a research project, it ranges from half an hour to 10 hours of work by someone from the team.*” ~ Group 2

“*We don’t speak the same language, that’s for sure. Even if you popularize a lot, it's not because you use words that I know that I understand what you're talking about. That’s a barrier for sure. It must first be popularized and perhaps schematized and made as simple as possible so that we know where we are going.*” ~ Group 3

Mitigating these barriers requires further promoting patient involvement in collaborations, implementing effective recruitment strategies, and disseminating scientific information in a language understandable to all.

“*As soon as we have patients involved in research, they want more, it's not complicated, they become our main advocates, our promoters, they talk to friends about it, which often brings us more patients.*” ~ Group 2

“*I am clearly interested by our role as researchers and how we, basic researchers, can ultimately contribute to making things more accessible. Most of us are teachers, so we have an important communication and education role within the framework of our university function, but which can also go beyond that I think.*” ~ Group 1

“*The presence of patient partners forces us to modify our approach to be more on the ground. When you have a patient-partner in a meeting, it raises awareness.*” ~ Group 3

To increase patient involvement, groups 2 and 3 urged the development of projects that directly concern patients. As a recruitment strategy, groups 1 and 2 advised involving family physicians, who share a strong therapeutic bond with patients, and family medicine groups interested in research. Group 3 stressed the need to further popularize and simplify scientific concepts by seeking patient input.

Success indicators of a collaborative platform

To successfully implement a collaboration, the vast majority advocated for a network structure per field of research, involving a strong leadership to provide clear direction to projects while being flexible enough to adapt to the needs identified by the various stakeholders. When asked more specifically what they considered as success indicators of a collaborative platform, all three groups stated the relevance, strength, and duration of collaborations, the number of projects implemented and completed, and the grants and funding obtained through the platform. Additional success indicators reported by the participants included improved patient recruitment and response (groups 1 and 2), the number of publications arising from the platform (groups 1 and 3), and user experience, satisfaction, and retention (groups 2 and 3).

## Discussion

Overall, barriers, enablers, and possible solutions identified during the focus groups concur with the common strategies for an impactful collaboration listed in our logic model: financial support, leadership, guidelines, human and material resources, and organization (Appendix A) [10]. Several barriers and enablers under each category were commonly shared, suggesting that although actors of each research pillar have their own areas of expertise and experiences, they are aware and sensitive to the realities and challenges faced by the other pillars. The main findings under each category of barriers and enablers and their implications are further discussed below.

Translational research project concretization

The discussions highlighted that while everyone was interested in participating in translational research projects, many wondered how such projects could be accomplished in a concrete and efficient manner. Concerns about the complexity and length of the process are legitimate given the stagnant pace at which research evidence translates into improved healthcare and services [8]. Several translational research models exist and a consensus has yet to be reached on a clear and universal definition, which further complexifies the application of the process as part of the everyday practices [20]. Despite these divergences, it is progressively agreed that translational research should be viewed as a continuous bidirectional cycle, where each pillar informs the others on research questions to address, rather than a linear, unidirectional process starting solely with basic science [21,22].

As voiced by the participants, for the collaborative platform to be deemed suitable for all actors involved, its implementation should focus mainly on efficiency, simplicity, and accessibility, with the use of clear guidelines and targeted research questions, and the creation of spaces to facilitate dynamic exchanges. These constitute key points that must be considered meticulously when building the foundations of the platform to ensure its relevance, practicality, and durability. Examples of successful collaborative models include the Community of Patients for Research (ComPaRe) platform, launched in 2018 in France [23], and the Translational Outcomes Project in Neurotrauma (TOP-NT), also established in 2018 in the United States [24]. The ComPaRe platform brings together public research teams and patients to answer research questions on chronic disease management, while the TOP-NT facilitates the collaboration between preclinical and clinical researchers to develop traumatic brain injury assessment tools. However, since they are deployed on a national scale, both platforms require significant annual public funding to maintain their activities.

Basic research applicability

History has repeatedly proven that basic science is indispensable to medical progress, bringing significant leaps in improving global health such as antibiotics, vaccination, and DNA sequencing. Nowadays, it is increasingly challenging to apply basic research discoveries to clinical and community settings despite significant investments in basic science [25,26]. In the development of molecular biomarkers for instance, which is central to translational research, the return on investment appears negligible. Despite numerous publications describing promising candidates, very few biomarkers successfully reach their clinical utility, partly due to insufficient collaborative teams and lack of clinical application relevance [27,28].

As mentioned previously, research waste is a real issue given the low rate of applicable scientific data [7]. A plausible reason for the misuse of research evidence lies in the evaluation of scientists’ productivity, which relies heavily on the number of publications and bibliometric scores to boost professional success and secure financial support [29]. This traditional method of evaluating researchers is anchored in mentalities and undermines the social responsibility of science. A paradigm shift from knowledge production to knowledge usability beyond academia must occur so that basic research quality is quantified based on its societal impact rather than individual productivity [30,31]. To achieve this transformation, the scientific community, including academia, politics, funding agencies, industry, and scientific journals, must consensually recognize the social responsibility of research and the need for a genuine two-way dialogue with the public to drive innovation [32].

Clinician availability and commitment

Clinicians indisputably play a central role in translational research and their involvement is crucial to translate basic research results into clinical applications. There are clear performance benefits for health organizations to value research activities such as lower patient mortality rates, higher levels of patient satisfaction, reduced staff turnover, improved staff satisfaction, and improved organizational effectiveness [33]. Despite these significant advantages, support for research initiatives and involvement of clinicians in research programs are often lacking.

Intensive exposure of clinicians to research early in their academic training is essential to change their perceptions and behavior towards research [34,35]. To revitalize the clinician-researcher career pipeline, providing long-term research training opportunities to young medical residents and promoting co-publications with their training supervisors are significant predictors in the decision to pursue a research career [36]. Other important environmental elements need to be addressed to improve clinicians’ research interest and commitment. These include a stable infrastructure that supports and assists with research activities, measures guaranteeing protected research time and financial compensation aligned with the level of effort invested in research, and the relevance and feasibility of study designs in their practice settings [37,38].

Patient involvement and recruitment

The benefits of patient involvement in research are undeniable. Their participation not only enables them to gain knowledge and empowerment, but also strengthens the relevance, quality, and value of research, and facilitates the dissemination of results to those concerned [39]. Prioritizing research topics that directly affect patients is paramount to encourage their participation in research and, thereby, ease recruitment. Among the standard recruitment methods, and as a recruitment strategy advised by the participants, referral by a physician is indeed the most cost-effective recruitment method [40]. Nonetheless, special considerations must be accounted for upfront when designing a study, from both the patient and physician perspectives, to avoid recruitment issues associated with complex, time-consuming, and under-budgeted studies. Indeed, patients and physicians are more inclined to participate in clear, comprehensible studies that require reasonable time and effort [41]. Along the same lines, the participants insisted on the need to disseminate scientific information in a language understandable to all, especially for patients. To establish a deeper connection and solidify the relationship between science and society, communication is key. Researchers must make a conscientious effort to engage patients and the public early to determine and co-create the best methods of communicating research to nonexpert audiences [42].

To support the shift from primarily researcher-driven to patient-driven research and ensure that studies focus on patient-identified priorities, initiatives such as the Centre for Engagement and Dissemination (CED) in the United Kingdom [43], Patient-Centered Outcomes Research Institute (PCORI) in the United States [44], and Strategies for Patient-Oriented Research (SPOR) in Canada [45] use holistic approaches by engaging patients and the public as partners in the research process. The James Lind Alliance (Southampton, United Kingdom) is another initiative that has developed a consultation and prioritization process to bring together patients, caregivers, and clinicians within a partnership to define health research priorities [46].

Strengths and limitations

As a strength of this study, the choice of the focus group methodology coupled with the recruitment of participants representing various disciplines allowed us to collect a broad range of points of view in an open, dynamic, and convivial setting. Overall, participants enjoyed the discussions and found the activity interesting. Nevertheless, the data should be interpreted in their context and generalized with caution to other groups or research domains given the small sample size of the study. Furthermore, the patients’ perspective was supported by a single patient partner in Group 3. Given that patient involvement was a pivotal discussion topic, it might have been beneficial to gather further experiences and perceptions from multiple patients. Equal numbers of women and men were included in the study, but not within each focus group, particularly in Group 1 where there were no female representatives. This unfortunately reflects the persistent underrepresentation of women in most scientific disciplines [47]. The results might have differed if each focus group had comprised a more equitable distribution of women and men.

## Conclusions

This study shed light on the points of view of the actors within the research-to-practice continuum, allowing us to identify key barriers and enablers within each pillar to implement a functional and dynamic collaborative platform. Participants’ desire to collaborate combined with the absence of a tool to foster efficient collaborations are indicators of the importance of our approach. Beyond the financial and technical needs to promote translational research, our findings also indicate that a culture change is necessary to acknowledge the strong interdependence between the research pillars, the influence they exert on each other, and their equal importance in contributing to the common goal of improving the well-being and health of the population. Creating a readily accessible collaborative platform to all actors across the research-to-practice continuum has the potential to drive research advances based on the needs of patients, clinicians, and the public, and thus facilitate their clinical application in a timelier manner to improve healthcare standards and services as well as population health outcomes.

## Figures and Tables

**Figure 1:**
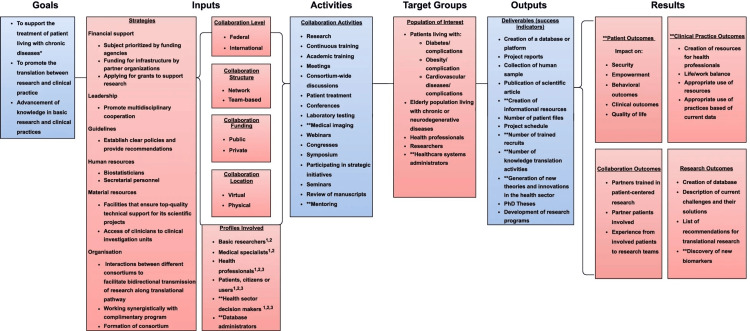
Logic model for a global translational research collaboration model *Diabetes, obesity, cardiovascular diseases, and their complications, and the elderly population living with chronic or neurodegenerative disease; **Elements that were not mentioned in the reviewed literature but were identified by experts in the field. 1,2,3 indicates profiles involved in the 1st, 2nd and 3rd pillars of research. Source: Lavertu et al., 2022 [10]; licensed under Creative Commons Attribution 4.0 International License, CC BY 4.0 Attribution 4.0 International

## Appendices

Appendix A: Logic model developed following scoping review on collaborative models

Appendix B: Facilitation guide for focus groups

Resources: 1 facilitator and 2 observers

Number of participants (Group 1): 8 basic researchers

Number of participants (Group 2): 8 clinical researchers

Number of participants (Group 3): 7 knowledge users (clinicians and patients)

Location: Virtual meeting via Zoom

Duration: 2 hours

Goals:

- Explore the perception of stakeholders

- Identify themes and methods of collaboration

Before the Start of the Meeting

Greeting (5 minutes):

- Welcome each participant warmly and dynamically

- Invite each participant to deactivate their microphone and reactivate it when they wish to speak

- Invite everyone to activate their camera to be visible to everyone

- Record attendance as participants arrive

Start the Meeting

Welcome remarks (3 minutes):

- Welcome participants

- Thank them for accepting the invitation. Mention that all the comments they express will greatly help the project

- Call on their participation and expertise

- Mention that this is an exchange of ideas, there are no right or wrong answers, we want to know their opinions and experiences

- Specify that the meeting will be recorded

- Before starting, ask participants if they have any questions or concerns

Presentation (5 minutes):

- Introduce yourself and all team members

- Explain your role, as well as that of other team members

- As moderator-presenter, you are responsible for:

o   Facilitating the meeting

o   Asking the questions

o   Managing speaking turns

o   Managing speaking time, everyone’s ideas and non-judgment

- For team members:

o   Note the essential elements that will emerge from the discussions

o   Record the meeting

Speaking turn (7 minutes):

- Take speaking turns so that participants can say their first and last name, their positions, and main affiliations.

Progress of the meeting (10 minutes):

- Present the agenda:

o   Explain the objective of establishing a collaboration platform

o   Feedback on the scoping review: Short presentation of the results obtained

o   Period of exchange and discussion

o   Conclusion (We expect the meeting to end at… o’clock)

o   Explain the objectives and criteria that guided the choice of participants

- Explain the rules:

o   Prompt to activate cameras and turn audio on/off as needed

o   Recording of the meeting for data analysis reasons

o   Confidentiality of comments: no comments made must leave the meeting, nor be taken up in another       context

o   There is no wrong answer, everything is important

o   It is best to speak one at a time

o   Respect for all opinions is mandatory

- Express what is expected of participants:

o   Let everyone have the chance to express themselves

o   They must welcome other comments without blame or judgment

o   Their comments must be authentic and expressed with respect

o   Discussions should lead to constructive solutions

o   Announce the maximum time allocated to the meeting: 2.5 hours maximum

- Present the predictable outcomes:

o   Collection of all comments expressed

o   Data analysis

o   Production of a scientific article

Questions for participants (maximum 80 minutes):

- Discussion between participants

- Use the series of questions in Appendix 2

Summary of discussions (5 minutes)

- Summarize the gist of the comments: what we remember from what participants expressed

Conclusion (3 minutes)

- Remind the predictable outcomes:

o   Collection of all comments expressed

o   Data analysis

o   Production of a scientific article (accessible to participants)

Acknowledgments (2 minutes)

- Express your gratitude to the participants for their involvement and comments

Appendix C: Questions to ask during focus groups

**Table 3:** Questions for basic researchers (Group 1) Reference for type of question: Krueger and Casey, 2000 [18]

Type of question [18]	Targeted information	Question	Time allocated
Opening question	Introduction from participants	Can you briefly introduce yourself and tell us how long you have been working in the field of basic research?	10 min (1 min per participant)
Introductory questions	Background and experience	Can you share some experiences you have in terms of collaboration in translational research?	10 min
		Translational research is a process that ensures the transition of basic research to its applications in humans. Do you know of any collaborative initiatives in translational research that bring together basic researchers and primary care medicine?	5 min
		What place does primary care occupy in your areas of research?	5 min
Transition question	Bring the main topic	What would you think of a collaboration between basic researchers and primary care clinical teams in the fight against chronic diseases? Share your first impression with us.	5 min
Key questions	Implementation	How do you see the implementation of such a collaborative initiative? Tell us about how it could work in practice.	5 min
	Operation	What is your perception of the operation of a collaborative platform?	5 min
		In your opinion, what are the most important elements to consider before, during and after starting a collaborative initiative?	5 min
	Barriers	What do you think would be the barriers to implement a collaborative platform?	5 min
	Enablers	In your opinion, what are the elements that could facilitate the implementation of such a collaborative initiative?	5 min
	Success	What would be the best ways to sustain such a collaborative initiative? Tell us about what you have observed in similar collaborations.	5 min
		What do you think would be a way to evaluate the success of a collaborative platform? Tell us about the success indicators you know or think about.	5-10 min
Closing Questions	Reviews, opinions and suggestions	Can you think of any other important aspects that were not covered today?	5-10 min
		Would you be interested in joining such a collaboration? If so, tell us about the role you see yourself playing. If not, tell us briefly about your reluctance or impediments.	5-10 min

**Table 4:** Questions for clinical and organizational researchers (Group 2) Reference for type of question: Krueger and Casey, 2000 [18]

Type of question [18]	Targeted information	Question	Time allocated
Opening question	Introduction from participants	Can you briefly introduce yourself and tell us how long you have been practicing in your field?	10 min (1 min per participant)
Introductory questions	Background and experience	Can you share some experiences you have in terms of collaboration in translational research?	10 min
		Translational research is a process that ensures the transition of basic research to its applications in humans. Do you know of any collaborative initiatives in translational research that bring together basic researchers and primary care medicine?	5 min
		What place do primary care and basic research occupy in your professional life?	5 min
Transition question	Bring the main topic	What would you think of a collaboration between basic researchers and primary care clinical teams in the fight against chronic diseases? Share your first impression with us.	5 min
Key questions	Implementation	How do you see the implementation of such a collaborative initiative? Tell us about how it could work in practice.	5 min
	Operation	What is your perception of the operation of a collaborative platform?	5 min
		In your opinion, what are the most important elements to consider before, during and after starting a collaborative initiative?	5 min
	Barriers	What do you think would be the barriers to implement a collaborative platform?	5 min
	Enablers	In your opinion, what are the elements that could facilitate the implementation of such a collaborative initiative?	5 min
	Success	What would be the best ways to sustain such a collaborative initiative? Tell us about what you have observed in similar collaborations.	5 min
		What do you think would be a way to evaluate the success of a collaborative platform? Tell us about the success indicators you know or think about.	5-10 min
Closing Questions	Reviews, opinions and suggestions	Can you think of any other important aspects that were not covered today?	5-10 min
		Would you be interested in joining such a collaboration? If so, tell us about the role you see yourself playing. If not, tell us briefly about your reluctance or impediments.	5-10 min

**Table 5:** Questions for knowledge users (Group 3) Reference for type of question: Krueger and Casey, 2000 [18]

Type of question [18]	Targeted information	Question	Time allocated
Opening question	Introduction from participants	Can you briefly introduce yourself and tell us how long you have been involved in primary care?	10 min (1 min per participant)
Introductory questions	Background and experience	Can you share some experiences you have in terms of collaboration in translational research?	10 min
		Translational research is a process that ensures the transition of basic research to its applications in humans. Do you know of any collaborative initiatives in translational research that bring together basic researchers and primary care medicine?	5 min
		What place does basic research occupy in your professional life?	5 min
Transition question	Bring the main topic	What would you think of a collaboration between basic researchers and primary care clinical teams in the fight against chronic diseases? Share your first impression with us.	5 min
Key questions	Implementation	How do you see the implementation of such a collaborative initiative? Tell us about how it could work in practice.	5 min
	Operation	What is your perception of the operation of a collaborative platform?	5 min
		In your opinion, what are the most important elements to consider before, during and after starting a collaborative initiative?	5 min
	Barriers	What do you think would be the barriers to implement a collaborative platform?	5 min
	Enablers	In your opinion, what are the elements that could facilitate the implementation of such a collaborative initiative?	5 min
	Success	What would be the best ways to sustain such a collaborative initiative? Tell us about what you have observed in similar collaborations.	5 min
		What do you think would be a way to evaluate the success of a collaborative platform? Tell us about the success indicators you know or think about.	5-10 min
Closing Questions	Reviews, opinions and suggestions	Can you think of any other important aspects that were not covered today?	5-10 min
		Would you be interested in joining such a collaboration? If so, tell us about the role you see yourself playing. If not, tell us briefly about your reluctance or impediments.	5-10 min
